# Caffeine intake improves the cognitive performance of patients with chronic kidney disease

**DOI:** 10.3389/fmed.2022.976244

**Published:** 2022-10-14

**Authors:** Linpei Jia, Hanxue Zhao, Lixiao Hao, Lin-Hui Jia, Rufu Jia, Hong-Liang Zhang

**Affiliations:** ^1^Department of Nephrology, Xuanwu Hospital, Capital Medical University, Beijing, China; ^2^College of Basic Medicine, Capital Medical University, Beijing, China; ^3^Department of General Medicine, Xuanwu Hospital, Capital Medical University, Beijing, China; ^4^College of Basic Medicine, Southern Medical University, Guangzhou, China; ^5^Administrative Office, Central Hospital of Cangzhou, Cangzhou, China; ^6^Department of Life Sciences, National Natural Science Foundation of China, Beijing, China

**Keywords:** caffeine, chronic kidney disease, cognitive performance, the National Health and Nutrition Examination Survey, cognitive impairment

## Abstract

**Objective:**

Cognitive impairment is a common complication of chronic kidney disease (CKD). Caffeine intake has been reported to improve cognitive performance in several studies. However, whether the benefits of caffeine intake on cognitive function apply to patients with CKD remains unknown.

**Methods:**

We performed a retrospective cross-sectional study based on the National Health and Nutrition Examination Survey (NHANES). The data of CKD subjects and non-CKD subjects from NHANES 2011−2014 were analyzed. Propensity score matching (PSM) was performed based on age, sex, diabetes, cancer, educational level, energy intake and protein intake to select subjects. The Consortium to Establish a Registry for Alzheimer’s Disease Word Learning Test (CERAD-WL), the CERAD Word List Recall Test (CERAD-DR), the Animal Fluency Test (AF) and the Digit Symbol Substitution Test (DSST) were used, whereby the occurrence of cognitive impairment was identified. Logistic regression models were performed to evaluate the association between caffeine intake and cognitive performance in CKD and non-CKD participants. Stratified analyses according to the stage of CKD and the urinary albumin/creatinine ratio levels were performed. Plot curves were then generalized to present a non-linear relationship, and the inflection point for each non-linear model was obtained by using a recursive algorithm.

**Results:**

Cognitive impairment was more prevalent in CKD patients than in non-CKD subjects. For CKD patients, caffeine intake was associated with higher CERAD-WL, CERAD-DR, AF and DSST scores. For non-CKD subjects, caffeine intake was associated with higher DSST scores only. Subgroup analysis revealed that caffeine only benefited the cognitive function of patients with CKD stages 2 and 3. The analysis showed non-linear relationships of caffeine intake and cognitive function for both CKD and non-CKD subjects. The inflection point of caffeine intake for CKD patients was 279 mg/day.

**Conclusion:**

The recommended dose of caffeine intake to improve the cognitive function of CKD patients is ≤279 mg/day.

## Introduction

Chronic kidney disease (CKD) poses a major challenge to public health due to its detrimental impact on individuals’ quality of life. Morbidity and mortality caused by CKD and CKD-related complications have increased drastically in recent years (1). Cognitive impairment is a common complication during the progression of CKD (2). It affects 27% to 62% of patients at CKD stages 1−4 (3). Various tests, including the Montreal Cognitive Assessment (MoCA), the Mini Mental State Examination (MMSE), the Trail-making Test, and the Consortium to Establish a Registry for Alzheimer’s disease (CERAD) are routinely used to assess the cognitive function of CKD patients (2, 4, 5). In developing countries, the morbidity of cognitive impairment in CKD patients reaches 50% or higher (6, 7). A decreased estimated glomerular filtration rate (eGFR) was found to be associated with cognitive dysfunction in several population-based cohorts (5, 8). Cognitive impairment remarkably decreases the living ability, quality of life and treatment compliance, which further increases the difficulty of treatment as well as the risk of mortality of CKD patients (9). Therefore, early identification and prevention of cognitive impairment in CKD patients are of great importance.

The exact mechanism for cognitive dysfunction in CKD remains unknown; however, multisystemic factors, including hypertension, anemia, acidosis, proteinuria, and uremic milieu, may conceivably exert a detrimental effect on the brains of patients with CKD (10).

Dietary patterns may positively affect cognitive performance, especially for the learning and memory abilities of individuals (11). Healthy dietary patterns such as the Mediterranean diet potentially reduce the risk of cognitive impairment such as in Alzheimer’s disease (12). However, nutrients or compounds implicated in healthy diets that benefit cognitive performance are still under investigation.

**FIGURE 1:**
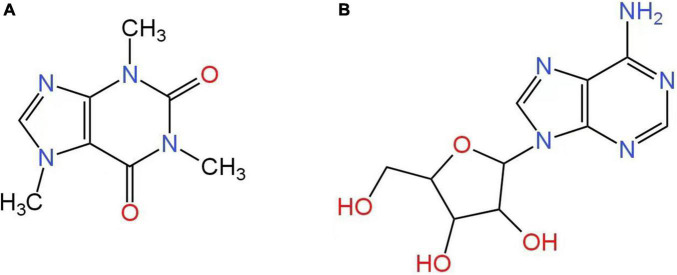
Chemical structures of caffeine and adenosine. The structure of caffeine **(A)** is similar to that of adenosine **(B)** due to the purine backbone.

Caffeine (1,3,7-trimethylxanthine), a purine alkaloid detectable in various drinks, including coffee, cola, and tea, is probably the most commonly used psychoactive substance/psychostimulant worldwide. During the COVID-19 pandemic, the consumption of caffeine, especially coffee and tea, has increased, partially because these beverages could reduce the accumulated stress of dramatic changes in lifestyle (13). Caffeine has a similar structure to adenosine (Figure 1) (14). As a nervous system stimulant, caffeine has been revealed by numerous studies to have an effect on cognitive functions, including memory and attention (15). Caffeine consumption may enhance processing speed and relieve anxiety (16, 17). For adults, caffeine may also jeopardize oral health, including increasing the risk of tooth loss (18). Caffeine may have a greater impact on overall health than just mental and brain health.

Diet is a fundamental component of CKD integrated treatments. A proper diet reduces the complications of and improves the prognosis of CKD (19). Higher caffeine consumption was associated with a lower risk of incident CKD, with each additional cup consumed lowering the risk of CKD by 3% (20), especially for adults with metabolic syndrome (21). Higher coffee consumption is also associated with lower all-cause mortality in patients with CKD (22). Moderate caffeine intake through habitual coffee consumption is beneficial to cognitive function in patients receiving hemodialysis, possibly due to selective enhancement of attention and vigilance (10). Notwithstanding, whether caffeine benefits patients with CKD in terms of cognitive impairment is unknown.

## Aim

In the present study, we aimed to examine the potential effect of caffeine intake on the cognitive performance of patients with CKD. We hypothesize that caffeine intake benefits patients with CKD in terms of alleviating cognitive impairment. While this hypothesis was being tested, we further sought to identify a beneficial dosage of caffeine. To this end, the relationships between caffeine intake and cognitive functions in CKD and non-CKD subjects were investigated based on a cross-sectional national study in the United States.

## Materials and methods

### Data source

The National Health and Nutrition Examination Survey (NHANES) is a cross-sectional national survey performed by the Centers for Disease Control and Prevention (CDC) of the United States every 2 years that aims to evaluate the health and nutritional status of the United States population^1^. Subjects of NHANES were sampled on the basis of a set of complicated, stratified and multistage study protocols. Then, demographic, dietary, body examination, laboratory, drug use, disease history and living habits data were collected either by interviews at home or health examinations in the mobile examination center (MEC). All NHNAES data are publicly available see text footnote 1. The protocols of NHANES were approved by the CDC of the United States, and written informed consent was obtained from all participants.

### Subject selection

Datasets of the 2011−2012 cycle and the 2013−2014 cycle were extracted. The total number of participants in these two cycles was 19931. A 24-h dietary recall interview was performed in each survey cycle. Because individuals aged 80 and over are top coded to ensure their anonymity, the specific ages of these individuals are not available^2^. Adults younger than 80 years old who participated in the cognitive questionnaires and kidney function evaluation were included. We excluded participants with diets that differed from their usual dietary habits on the day before the interview. Subjects who were prescribed a special diet with energy intakes of <500 or >8000 kcal/day for men and <500 or >5000 kcal/day for women were excluded (23). CKD was defined as an estimated glomerular filtration rate (eGFR) < 60 mL/min/1.73 m^2^ and/or a urinary albumin creatinine ratio (ACR) >30 mg/g (24). eGFR was calculated by the Chronic Kidney Disease Epidemiology Collaboration equation (25). Participants without weight or ACR data were excluded.

### Collection of caffeine intake and dietary data

Based on the 24-h dietary recall interview in each survey cycle, caffeine consumption was estimated from the foods and beverages that contain caffeine by the survey called What We Eat in America (26). Dietary status was collected using an in-person 24-h recall by a computerized 5-step method and a second non-consecutive 24-h recall *via* telephone. Data for each nutrient were calculated (27). We extracted the total energy, protein, carbohydrate, sugar and fiber intake data from the database.

### Cognitive function evaluation

Four scale assessments were performed in NHANES, including the Consortium to Establish a Registry for Alzheimer’s Disease Word Learning Test (CERAD-WL), the Consortium to Establish a Registry for Alzheimer’s Disease Word List Recall Test (CERAD-DR), the Animal Fluency test (AF), and the Digit Symbol Substitution test (DSST) (28). The CERAD-WL and CERAD-DR were used to evaluate immediate and delayed verbal memory, respectively (29). The AF tested language ability and executive function (30). The DSST was used to assess cognitive executive function and processing speed (31). The detailed process of each test was described in the NHANES protocol^3^ as well as in previous studies (32). The cutoff values of the tests for low cognitive performance were set as CERAD-WL < 17, CERAD-DR < 5, AF < 14, or DSST < 40 (33). The relationship between caffeine intake and the test scores was investigated.

### Propensity score matching and the matching factors

To identify the confounding factors, we adjusted the logistic regression model by covariates. Accounting for the baseline differences, propensity score matching (PSM) was performed to investigate the effects of caffeine intake on cognitive function. The matching conditions included age, BMI, sex, diabetes, cardiovascular disease, cancer, educational level, energy intake and protein intake. Demographic data, including age, sex and education status, were extracted from the NHANES database. The BMI value was calculated by dividing the weight (kg) by the height squared (m^2^). The medical history of the patients (diabetes, cardiovascular disease and cancer) and daily nutrient intakes (total energy and protein) were collected by questionnaires.

### Statistics

Statistical analysis was performed by Empower Stats^4^. In NHANES, all data were weighted according to the analytical guidelines of the NHANES^5^ for better representation of the total. For continuous variables, medians are expressed and compared by *Student’s t*-test. For categorical variables, data are expressed as percentages and analyzed by the chi-squared test. Logistic regression analysis was performed to evaluate the association between caffeine intake and cognitive performance in non-CKD and CKD participants. Subgroup analysis was performed according to the different CKD stages and ACR levels. CKD patients were divided into a CKD stage 1 group, CKD stage 2 and 3 groups and CKD stage 4 and 5 groups. According to the ACR levels, CKD patients were grouped into ACR < 30 mg/mmol, 30 mg/mmol ≤ ACR < 300 mg/mmol and ACR ≥ 300 mg/mmol groups. Logistic regressions of caffeine intake and cognitive function were performed in each subgroup. Graphics were generate to show the non-linear relationship. The inflection point was calculated for each non-linear model using a recursive algorithm in which a two-piecewise linear regression model was adopted on both sides (34).

## Results

### Demographic data of the studied subjects

**FIGURE 2:**
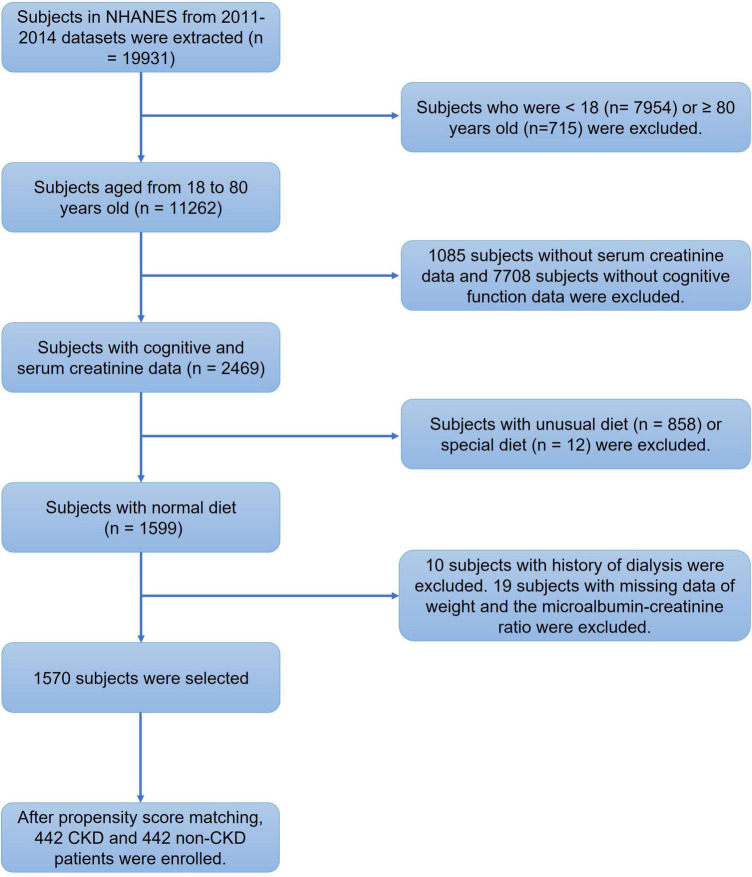
Flowchart of the screening process for eligible subjects. We extracted the clinical data of 19931 subjects from the National Health and Nutrition Examination Survey (NHANES) 2011–2014 database. We first excluded 7954 subjects under 18 years old and 715 subjects aged more than 80 years old. Then, 8793 subjects with missing serum creatinine or cognitive evaluation data were excluded. We also excluded 870 subjects with special diets. Among the remaining 1599 subjects, 10 subjects with a dialysis history and 19 subjects without diagnostic prerequisites for chronic kidney disease (CKD) were excluded. Then, 1570 subjects were enrolled for analysis. After propensity score matching, 442 CKD patients and 442 non-CKD subjects were finally enrolled.

Among the 19931 subjects in NHANES 2011−2014, 1570 subjects were enrolled after excluding 18361 subjects based on the exclusion criteria (Figure 2). PSM selected 442 non-CKD subjects and 442 CKD patients from the matching criteria (Figure 2). Only subjects aged over 60 years old were tested for cognitive performance in NHANES, and therefore, the mean age of the enrolled subjects was 68.68 (*SD* = 5.63) years old. Men accounted for 53.39% of all subjects. Whites were the major ethnicity, accounting for 47.06%. The mean age of the CKD patients was 69.21 (*SD* = 5.63) years old, which was higher than that of the non-CKD subjects with a mean age of 68.15 (*SD* = 5.60) years old (Table 1, *P* = 0.005). No ethnic difference was found between the non-CKD and CKD subgroups (Table 1, *P* = 0.261 for ethnicities); thus, the intergroup comparisons were conducted without regard to ethnicity.

**TABLE 1:** Clinical characteristics of subjects in National Health and Nutrition Examination Survey (NHANES) 2011−2014.

Clinical variables	Total (*N* = 884)	Non-CKD (*N* = 442)	CKD (*N* = 442)	*P*	Effect size
Age (year)	68.68 ± 5.63	68.15 ± 5.60	69.21 ± 5.63	0.005	0.189
Male (%)	53.39	54.75%	52.04%	0.418	0.055
**Race**				0.261	0.014
White (%)	47.06	45.48	48.64		
Black (%)	24.43	23.08	25.79		
Other races (%)	28.51	31.44	25.75		
**Education**				0.700	−0.053
Less than 9th grade (%)	11.54	11.76	11.31		
9−11th grade (%)	14.37	13.57	15.16		
High school graduate (%)	21.83	21.27	22.40		
College or AA degree (%)	29.75	28.96	30.54		
College graduate or above (%)	22.51	24.43	20.59		
Body Mass Index (kg/m^2^)	29.84 ± 6.78	29.51 ± 6.82	30.17 ± 6.73	0.148	0.097
Systolic blood pressure (mmHg)	132.47 ± 18.76	130.41 ± 17.57	134.52 ± 19.69	0.001	0.226
Diastolic blood pressure (mmHg)	67.88 ± 14.43	67.96 ± 13.68	67.81 ± 15.15	0.740	−0.011
Pulse (bpm)	70.66 ± 11.96	70.40 ± 11.44	70.92 ± 12.46	0.522	0.044
Alanine transaminase (U/L)	22.26 ± 9.83	22.78 ± 9.22	21.74 ± 10.38	0.115	−0.106
Aspartate aminotransferase (U/L)	25.12 ± 9.66	24.98 ± 9.48	25.25 ± 9.85	0.679	0.028
Alkaline phosphatase (U/L)	69.65 ± 22.52	67.52 ± 18.81	71.79 ± 25.55	0.005	0.190
γ-glutamyl transferase (U/L)	27.31 ± 28.78	26.29 ± 26.19	28.33 ± 31.15	0.294	0.071
Blood urea nitrogen (mmol/L)	5.87 ± 2.38	5.01 ± 1.52	6.73 ± 2.75	<0.001	0.774
Total cholesterol (mmol/L)	4.90 ± 1.14	4.98 ± 1.08	4.82 ± 1.18	0.028	−0.142
Lactate dehydrogenase (U/L)	132.49 ± 27.42	128.76 ± 22.42	136.22 ± 31.22	<0.001	0.275
Creatine phosphokinase (IU/L)	134.61 ± 99.01	134.66 ± 92.21	134.57 ± 105.48	0.989	−0.001
Total protein (g/L)	71.07 ± 4.98	70.79 ± 4.84	71.34 ± 5.10	0.100	0.111
Uric acid (μmol/L)	351.19 ± 88.02	326.48 ± 75.22	375.91 ± 92.89	<0.001	0.585
Blood glucose (mmol/L)	6.49 ± 2.90	6.23 ± 2.55	6.74 ± 3.19	0.009	0.177
Serum phosphorus (mmol/L)	1.21 ± 0.18	1.20 ± 0.17	1.23 ± 0.19	0.008	0.166
Serum sodium (mmol/L)	139.51 ± 2.51	139.63 ± 2.51	139.40 ± 2.51	0.172	−0.092
Serum potassium (mmol/L)	4.06 ± 0.41	4.02 ± 0.37	4.10 ± 0.44	0.003	0.197
Serum iron (μmol/L)	14.84 ± 5.55	15.31 ± 5.56	14.36 ± 5.49	0.011	−0.011
Serum calcium (mmol/L)	2.37 ± 0.09	2.36 ± 0.09	2.38 ± 0.10	0.007	0.210
Hemoglobin (g/dL)	13.85 ± 1.43	13.99 ± 1.30	13.71 ± 1.55	0.003	−0.196
Caffeine intake (mg/day)	153.54 ± 183.38	154.40 ± 190.42	152.69 ± 176.28	0.890	−0.009
Energy intake (kcal/day)	1858.78 ± 782.73	1939.11 ± 827.57	1778.44 ± 727.27	0.002	−0.206
Protein intake (g/day)	74.77 ± 36.64	78.21 ± 40.31	71.34 ± 32.25	0.005	−0.188
Carbohydrate intake (g/day)	224.03 ± 99.71	236.16 ± 104.80	211.91 ± 92.90	<0.001	−0.245
Sugar intake (g/day)	93.90 ± 59.22	98.77 ± 60.73	89.03 ± 57.32	0.014	−0.165
Fiber intake (g/day)	17.25 ± 10.88	19.09 ± 12.30	15.42 ± 8.89	<0.001	−0.342
Coronary heart disease (%)	8.82	6.79	10.86	0.036	0.141
Stroke (%)	7.13	5.66	8.60	0.145	0.098
Cancer (%)	18.33	18.78	17.87	0.941	0.005
Diabetes (%)	29.41	23.76	35.07	0.001	0.167
CERAD-WL < 17	25.97	23.45	28.47	0.090	0.114
CERAD-DR < 5	22.36	19.63	25.06	0.055	0.129
AF < 14	25.55	22.63	28.44	0.050	0.132
DSST < 40	32.59	28.14	37.12	0.005	0.190

AF, Animal Fluency test; CERAD-WL, Consortium to Establish a Registry for Alzheimer’s Disease Word Learning test; CERAD-DR, Consortium to Establish a Registry for Alzheimer’s Disease Delayed Recall test; DSST, Digit Symbol Substitution test.

### Higher prevalence of cognitive impairment in chronic kidney disease patients than in non-chronic kidney disease subjects

We compared the demographic data, laboratory tests, disease history, dietary intakes and cognitive performance of CKD and non-CKD subjects. The gender composition (*P* = 0.418), education status (*P* = 0.700) and caffeine consumption (*P* = 0.890) showed insignificant differences between the two groups (Table 1). Compared with non-CKD subjects, the systolic blood pressure (SBP, *P* < 0.001) of CKD patients was much higher (Table 1). The diastolic blood pressure showed no difference between the CKD and non-CKD patients (*P* = 0.740). The variances in the kidney function (blood urea nitrogen and uric acid), liver function (alkaline phosphatase and lactate dehydrogenase) and ion levels (serum phosphorus, potassium, iron and calcium) coincided with the disease characteristics of CKD. The comparison of dietary intake showed that CKD patients consumed diets with lower levels of energy (*P* = 0.002), protein (*P* = 0.005), carbohydrate (*P* < 0.001), sugar (*P* = 0.014) and fiber (*P* < 0.001). Regarding complications, the comorbidity rates of coronary heart disease (*P* = 0.036) and diabetes (*P* = 0.001) were higher in CKD patients than in non-CKD subjects. For CKD patients, ratios of cognitive impairment in DSST (*P* = 0.005) were higher than in non-CKD subjects, which means CKD patients were more predisposed to cognitive executive function and processing speed impairment. Other cognitive parameters, including CERAD-WL (*P* = 0.090), CERAD-DR (*P* = 0.055) and AF (*P* = 0.050), showed no difference between CKD and non-CKD patients.

### Promoting effect of caffeine on the cognitive performance of chronic kidney disease patients and non-chronic kidney disease subjects

Logistic regression analysis was used to investigate the relationship between each cognitive test and caffeine consumption. Caffeine was confirmed to be associated with an improved cognitive performance in all enrolled subjects (Table 2). Subgroup analysis was performed for the CKD and non-CKD subjects. For the CKD patients, CERAD-WL, AF and DSST were significantly associated with caffeine consumption in the unadjusted model (Table 2, *P* < 0.050). However, caffeine only improved the performance on the DSST for non-CKD patients (Table 2, *P* = 0.022). For the CERAD-DR test, caffeine consumption did not affect the cognitive function of either non-CKD or CKD subjects.

**TABLE 2:** Logistic regression analysis for non- chronic kidney disease (CKD) and CKD subjects between each cognitive test and caffeine intake.

	Non-CKD (*N* = 442)		CKD (*N* = 442)		Total (*N* = 884)	
	OR (95%CI)	*P*-value	OR (95%CI)	*P*-value	OR (95%CI)	*P*-value
CERAD-WL	0.001 (−0.001, 0.003)	0.311	0.003 (0.001, 0.005)	0.010	0.002 (0.000, 0.003)	0.013
CERAD-DR	0.001 (−0.000, 0.002)	0.083	0.001 (−0.000, 0.002)	0.073	0.001 (0.000, 0.002)	0.013
AF	0.002 (−0.001, 0.004)	0.183	0.004 (0.001, 0.007)	0.003	0.003 (0.001, 0.005)	0.003
DSST	0.009 (0.001, 0.016)	0.022	0.014 (0.006, 0.022)	<0.001	0.011 (0.006, 0.017)	<0.001

AF, Animal Fluency test; CERAD-WL, Consortium to Establish a Registry for Alzheimer’s Disease Word Learning test; CERAD-DR, Consortium to Establish a Registry for Alzheimer’s Disease Delayed Recall test; DSST, Digit Symbol Substitution test.

### Caffeine intake specifically benefits the cognitive function of patients with stage 2 and 3 chronic kidney disease

**FIGURE 3:**
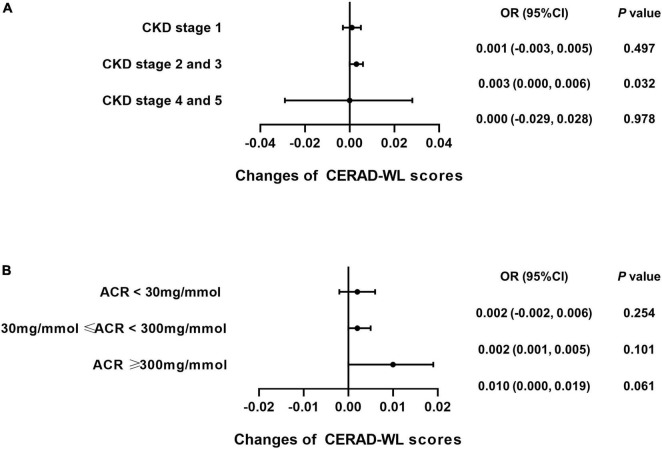
Caffeine intake improves performance on the Consortium to Establish a Registry for Alzheimer’s Disease Word Learning Test (CERAD-WL) for CKD stage 2 and 3 patients. To investigate whether CKD stages and urinary albumin creatinine ratio (ACR) levels influence the beneficial effects of caffeine intake on CERAD-WL in CKD patients, subgroup analysis was performed. Logistic regression analysis of caffeine intake and CERAD-WL scores was performed in each CKD stage subgroup (CKD stage 1 = 59 subjects, CKD stage 2 and 3 = 362 subjects, CKD stage 4 and 5 = 21 subjects) or each ACR subgroup (ACR < 30 mg/mmol = 196 subjects, 30 mg/mmol ≤ ACR < 300 mg/mmol = 210 subjects, ACR ≥ 300 mg/mmol = 36 subjects). Caffeine intake significantly improved the performance on the CERAD-WL in CKD stage 2 and 3 patients only (OR = 0.003, 95% CI: 0.000–0.006, *P* = 0.032, **A**). Caffeine did not affect the CERAD-WL score in any ACR subgroup **(B)**.

**FIGURE 4:**
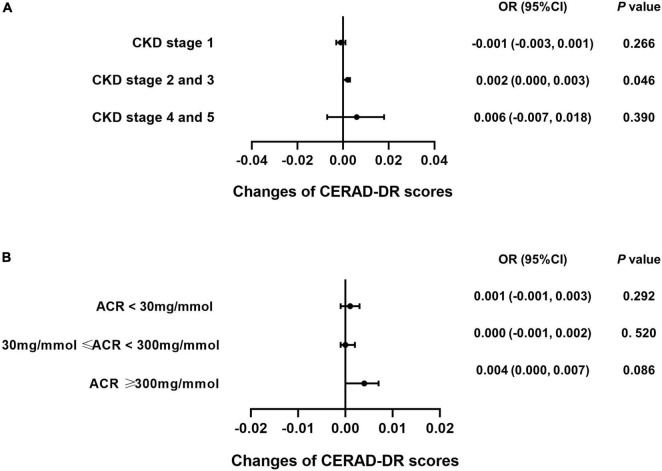
Caffeine only improves the results of the Consortium to Establish a Registry for Alzheimer’s Disease Word List Recall Test (CERAD-DR) for CKD stage 2 and 3 patients. Logistic regression analysis of caffeine intake and CERAD-DR scores was performed for the CKD stage (CKD stage 1 = 59 subjects, CKD stage 2 and 3 = 362 subjects, CKD stage 4 and 5 = 21 subjects) or urinary albumin creatinine ratio (ACR) subgroups (ACR < 30 mg/mmol = 196 subjects, 30 mg/mmol ≤ ACR < 300 mg/mmol = 210 subjects, ACR ≥ 300 mg/mmol = 36 subjects). Subgroup analysis showed that caffeine improved the results on the CERAD-DR in CKD stage 2 and 3 patients only (OR = 0.002, 95% CI: 0.000–0.003, *P* = 0.046, **A**). No beneficial effects were found among the ACR subgroups **(B)**.

**FIGURE 5:**
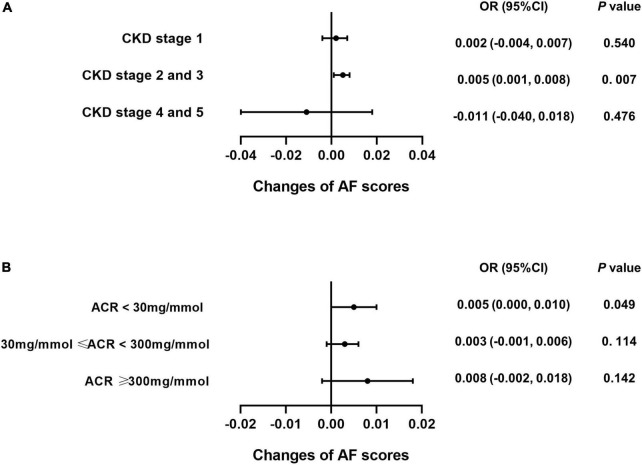
Caffeine benefits the performance on the Animal Fluency Test (AF) in CKD stage 2 and 3 patients. We performed logistic regression analysis of caffeine intake and AF scores in each CKD stage subgroup (CKD stage 1 = 59 subjects, CKD stage 2 and 3 = 362 subjects, CKD stage 4 and 5 = 21 subjects) and urinary albumin creatinine ratio (ACR) subgroup (ACR < 30 mg/mmol = 196 subjects, 30 mg/mmol ≤ ACR < 300 mg/mmol = 210 subjects, ACR ≥ 300 mg/mmol = 36 subjects). Forest plots show the odds ratios of all subgroups. For CKD stage 2 and 3 patients, caffeine intake significantly improved the AF scores (OR = 0.005, 95% CI: 0.001–0.008, *P* = 0.007, **A**). For CKD stage 1, 4 and 5 patients, caffeine did not improve the AF scores **(A)**. Among the ACR groups, only patients with an ACR less than 30 mg/mmol benefited from caffeine intake (OR = 0.005, 95% CI: 0.000–0.010, *P* = 0.049, **B**).

**FIGURE 6:**
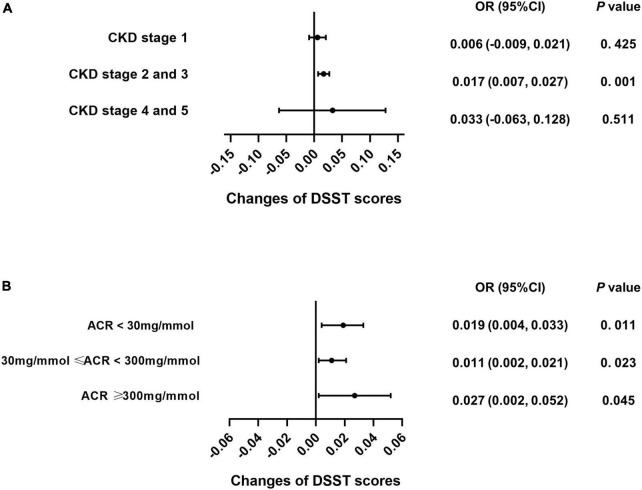
Caffeine improves scores on the Digit Symbol Substitution Test (DSST) for all CKD stages and ACR levels. We used logistic regression analysis to investigate whether caffeine intake would affect the DSST scores in each CKD subgroup (CKD stage 1 = 59 subjects, CKD stage 2 and 3 = 362 subjects, CKD stage 4 and 5 = 21 subjects) and urinary albumin creatinine ratio (ACR) subgroup (ACR < 30 mg/mmol = 196 subjects, 30 mg/mmol ≤ ACR < 300 mg/mmol = 210 subjects, ACR ≥ 300 mg/mmol = 36 subjects). Caffeine intake improved the performance of patients on the DSST with CKD stages 2 and 3 only (OR = 0.017, 95% CI: 0.007–0.027, *P* < 0.001, **A**). Caffeine benefited DSST for all ACR groups (ACR < 30 mg/mmol, OR = 0.019, 95% CI: 0.004–0.033, *P* = 0.011; 30 mg/mmol ≤ ACR < 300 mg/mmol, OR = 0.011, 95% CI: 0.002–0.021, *P* = 0.023; ACR ≥ 300 mg/mmol, OR = 0.027, 95% CI: 0.002–0.052, *P* = 0.045, **B**).

Subgroup analysis was performed for each test among the CKD patients to explore whether caffeine intake would benefit cognitive function in different CKD stages and ACR levels. The results revealed that caffeine consumption reduced the risk of cognitive impairment in patients with CKD stages 2 and 3 for all tests (**CERAD-WL**, OR = 0.003, 95% CI: 0.000−0.006, *P* = 0.032, Figure 3A; **CERAD-DR**, OR = 0.002, 95% CI: 0.000−0.003, *P* = 0.046, Figure 4A; **AF**, OR = 0.005, 95% CI: 0.001−0.008, *P* = 0.007, Figure 5A; **DSST**, OR = 0.017, 95% CI: 0.007−0.027, *P* = 0.001, Figure 6A). For ACR, caffeine consumption only influenced the risk of cognitive impairment in the ACR < 30 mg/mmol group of AF (OR = 0.005, 95% CI: 0.000−0.010, *P* = 0.049, Figure 5B) and all ACR groups of DSST (ACR < 30 mg/mmol, OR = 0.019, 95% CI: 0.004−0.033, *P* = 0.010; 30 mg/mmol ≤ ACR < 300 mg/mmol, OR = 0.011, 95% CI: 0.002−0.021, *P* = 0.023; ACR ≥ 300 mg/mmol, OR = 0.027, 95% CI: 0.002−0.052, *P* = 0.045, Figure 6B). Caffeine intake would not influcence cognitive performance in all ACR groups of CERAD-WL and CERAD-DR (Figures 3B, 4B).

### Moderate caffeine intake improved cognitive performance for both the non-chronic kidney disease and chronic kidney disease subjects

**FIGURE 7:**
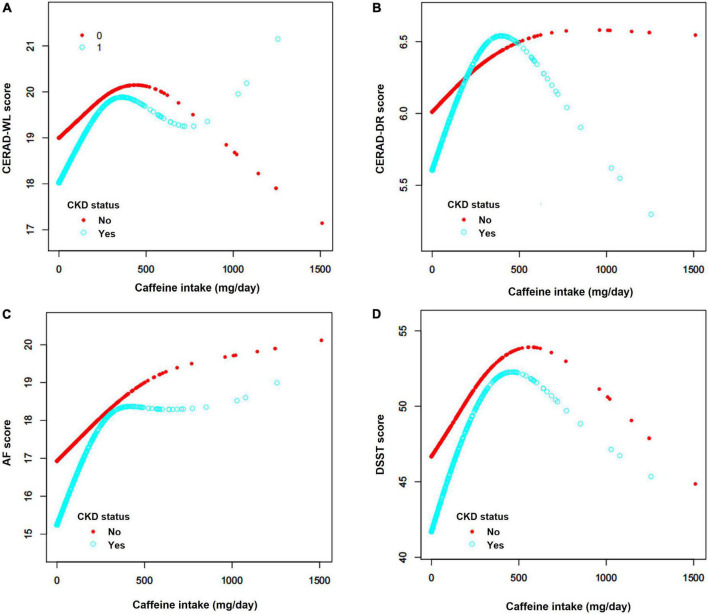
**(A–D)** Associations between caffeine intake and cognitive tests. The analyses showed a non-linear relationship between caffeine intake and the four cognitive tests, including the Consortium to Establish a Registry for Alzheimer’s Disease Word Learning Test (CERAD-WL), the Consortium to Establish a Registry for Alzheimer’s Disease Word List Recall Test (CERAD-DR), the Animal Fluency test (AF) and the Digit Symbol Substitution test (DSST). The blue curves represent CKD patients, and the red curves represent non-CKD subjects. For all tests, both of CKD and non-CKD subjects, positive associations were found when caffeine intake was below or equal to the inflection point. Negative associations were only found for the AF and DSST scores of CKD patients when caffeine intake was above the inflection point. The inflection points of CKD patients were 288 mg/day for CERAD-WL, 306 mg/day for CERAD-DR, 279 mg/day for AF and 294 mg/day for DSST.

Smooth curve fittings were generalized for non-CKD and CKD subjects to intuitively show the association between each cognitive test and caffeine consumption (Figure 7 and Table 3). A non-linear relationship was found for all cognitive tests for the two groups. The inflection points were determined with a two-piecewise linear regression model (Table 3). For CKD subjects, positive associations were shown when caffeine intake ≤ inflection points (*P* < 0.050), which means moderate caffeine intake would improve the cognitive performance of CKD patients. For non-CKD subjects, positive associations were observed in CERAD-WL and DSST when caffeine intake ≤ inflection points (*P* < 0.050). Thus, a moderate amount of caffeine consumption would improve immediate verbal memory, cognitive executive function and processing speed. When caffeine intake was above the inflection point, negative associations were found in the DSST (*P* = 0.027) of CKD patients. CKD patients with caffeine intake ≤ 279 mg/day benefited in terms of cognitive function (Table 3).

**TABLE 3:** Threshold effect analysis of each cognitive test and caffeine intake in Non-CKD and CKD subjects using the two-piecewise linear regression model.

	Non-CKD (*N* = 442)		CKD (*N* = 442)	
	OR (95%CI)	*P*-value	OR (95%CI)	*P*-value
**CERAD-WL**				
Inflection point (K)	304		288	
Caffeine intake < K	0.006 (0.002, 0.009)	0.003	0.007 (0.003, 0.011)	0.014
Caffeine intake > K	−0.003 (−0.006, 0.000)	0.085	−0.001 (−0.005, 0.003)	0.687
Log likelihood ratio	0.005		0.029	
**CERAD-DR**				
Inflection point (K)	67		306	
Caffeine intake < K	0.008 (−0.001, 0.017)	0.074	0.003 (0.001, 0.005)	0.004
Caffeine intake > K	0.000 (−0.001, 0.002)	0.509	−0.001 (−0.003, 0.001)	0.332
Log likelihood ratio	0.106		0.023	
**AF**				
Inflection point (K)	463		279	
Caffeine intake < K	0.003 (−0.000, 0.007)	0.085	0.013 (0.008, 0.019)	<0.001
Caffeine intake > K	−0.001 (−0.007, 0.004)	0.674	−0.004 (−0.009, 0.001)	0.089
Log likelihood ratio	0.261		<0.001	
**DSST**				
Inflection point (K)	463		294	
Caffeine intake < K	0.020 (0.009, 0.030)	<0.001	0.046 (0.032, 0.061)	<0.001
Caffeine intake > K	−0.012 (−0.029, 0.005)	0.163	−0.016 (−0.030, −0.002)	0.027
Log likelihood ratio	0.007		<0.001	

AF, Animal Fluency test; CERAD-WL, Consortium to Establish a Registry for Alzheimer’s Disease Word Learning test; CERAD-DR, Consortium to Establish a Registry for Alzheimer’s Disease Delayed Recall test; DSST, Digit Symbol Substitution test.

## Discussion

In this study, we investigated the association between caffeine intake and cognitive function in CKD patients. Our results show that higher levels of caffeine consumption benefited CKD patients on all cognitive tests except for the CERAD-DR and benefited the non-CKD subjects only on the DSST. When caffeine intake ≤ 279 mg/day for CKD patients, a linear dose–response association was detected between caffeine intake and cognitive performance.

In NHANES 2011−2014, the ratios of cognitive impairment in four cognitive function tests were all higher in CKD patients. However, the basis of cognitive impairment in patients with CKD is not clear. The possible mechanisms, however, are discussed as follows. First, CKD and cognitive impairment always share the same risk factors, including hypertension, diabetes, and smoking (35), which may result in the coexistence of these two diseases. Another possibility is that vascular changes in CKD (36), such as vascular calcification, epithelial dysfunction and arteriosclerosis, contribute to the insufficient blood supply to the brain and neuronal cell damage (37, 38). Likewise, the decreased clearance function of CKD leads to the accumulation of uremic neurotoxins, which may act on the kidney-brain axis and cause cognitive dysfunction (39). As such, patients with CKD may face a higher risk of cognitive impairment, and CKD has been identified as a risk factor for cognitive impairment, especially for vascular dementia.

Previous studies have explored the association between caffeine consumption and cognitive function in community-dwelling people. In the Prevencion con Dieta Mediterranea plus (PREDIMED-plus) study, researchers observed that dietary caffeine consumption was related to better cognitive function in a Mediterranean elderly population (40). Another study based on the NHANES 2011−2014 database also indicated that caffeine intake would benefit cognitive performance (23). Our findings that caffeine intake significantly improved the cognitive function of community-dwelling adults are consistent with previous studies.

The mechanism of action of caffeine on cognitive function is complex. As a central nervous system stimulant, caffeine could benefit cognitive function by three mechanisms (14). The first mechanism of caffeine could be described as a non-selective antagonist of adenosine receptors, which triggers the release of neurotransmitters, such as dopamine, norepinephrine, acetylcholine, gamma-aminobutyric acid, and glutamate (41). All of the abovementioned neurotransmitters can influence cognitive function. Methylxanthine, which is an organic molecule chemically similar to caffeine, can also improve the mobilization of intercellular calcium and inhibit phosphodiesterases, thereby enhancing cognitive function (42). Furthermore, caffeine could suppress neuroinflammation and act as a neuroprotectant by regulating the levels of protein kinase A and the blood supply in the nervous system (43).

We found that dietary caffeine intake improved scores on 3 of 4 cognitive tests for CKD patients. Caffeine may prevent afferent arteriolar constriction and increase renal plasma flow, which would accelerate the metabolism of uremic toxin (21). Although few studies have focused on the effects of caffeine on the cognitive function of CKD patients, caffeine was confirmed to be beneficial to CKD patients in decreasing the risk of CKD and improving their prognosis. Daily coffee intake was associated with a decreased risk of developing CKD (44). Similarly, another NHANES study also revealed an inverse association between caffeine consumption and all-cause mortality based on data from 4863 non-institutionalized American adults with CKD (45). Caffeine intake was dose-dependently associated with a lower incidence of CKD and albuminuria (22). The potential mechanism might be the adenosine antagonism of caffeine, which may prevent the constriction of glomerulus afferent arterioles (21), increase renal tissue oxygenation (46) and prevent renal fibrosis (47). However, the mechanism conferring a positive effect on kidney function needs further investigation.

In regard to the significantly positive effect on the cognitive function of CKD patients, direct evidence is not well documented in the literature. One possible explanation is that caffeine may reduce the incidence of depression, which is more common in patients with CKD and is closely associated with cognitive impairment (48, 49). Recent studies revealed that caffeine-derived metabolites could modulate the gut microbiota and improve depression (50). Collectively, caffeine may indirectly improve cognitive function by ameliorating renal function as well as directly stimulating the nervous system.

The subgroup analysis according to the stage of CKD showed that the beneficial effects of caffeine on cognitive function were only present in the CKD stage 2 and 3 groups. For patients with CKD stages 4 and 5, the clearance capacity of the kidney is severely damaged, which may cause the accumulation of caffeine metabolites in the blood. Higher levels of metabolites, which are equivalent to consuming a large amount of caffeine, are harmful to the nervous system. According to our results, the levels of ACR did not appear to act as an influencing factor on caffeine consumption and cognitive function in CKD patients. Thus, CKD stage 1 patients who have high ACR levels and a normal eGFR are not affected by caffeine.

Usually, a low caffeine intake refers to below 200 mg/day. Moderate caffeine intake is considered to be between 200 and 400 mg/day and high for above 400 mg/day (51). The recommended dose of caffeine intake for the general population is no more than 400 mg/day according to the European Food Safety Authority (52) and U.S. Food and Drug Administration (53). A daily dose of caffeine intake >500–600 mg may affect the cardiovascular system and nervous system by positive inotropic and anxiogenic-like effects leading to tachycardia, anxiety and so on, which increases the risk of health problems (53, 54). A dose of caffeine for cognitive protection or renal protection has not been recommended. A paucity of studies revealed that caffeine intake of 300−400 mg/day may enhance cognitive performance (23, 55). The morbidity and mortality of CKD patients would significantly decrease for populations with a caffeine intake of 200−400 mg/day. Caffeine may act as a methylxanthine compound to promote the movement of intercellular calcium (14). The proper concentrations of methylxanthine increase the uptake of calcium and neurotransmitters to affect the nervous system (14). However, higher concentrations of methylxanthine can inhibit the uptake of calcium. Our research further supports the efficacy of an appropriate dose of caffeine to protect cognitive function in CKD patients. When the dose was ≤ 279 mg/day, more caffeine intake was associated with better cognitive function. The recommended dose of caffeine intake was ≤ 279 mg/day for better cognitive function in CKD patients.

We acknowledge the limitations of this study. First, NHANES is not representative of all populations because of the ethnic composition of the United States, which limits the extrapolation of our findings to a more general population. Second, basic kidney function and cognitive status before caffeine consumption were unavailable. As a consequence, it remains unclear whether there was a direct cause-and-effect relationship between caffeine intake and kidney function. Further longitudinal studies are warranted to demonstrate the causal relationship between caffeine intake and cognitive function in CKD patients. Third, retrospective studies inevitably cause some bias. For example, the 24-h recall dietary interview by telephone may result in recall bias. Another important indicator, tobacco habits, was not included in this study because the smoking data of two-thirds of the subjects were missing from the NHANES datasets. A prospective study is expected in the future. Finally, only four cognitive tests were used in the NHANES database. Other commonly used cognitive tests, such as the Mini-mental State Examination and Montreal Cognitive Assessment, should be included in future studies for a more comprehensive assessment of cognitive function.

## Conclusion

Better cognitive performance is associated with daily caffeine consumption among community-dwelling people. Caffeine consumption confers a remarkable mitigating effect on the cognitive dysfunction of CKD patients. The recommended dose of caffeine intake should be no more than 279 mg/day for these patients.

## Data availability statement

The original contributions presented in this study are included in the article/supplementary material, further inquiries can be directed to the corresponding author/s.

## Author contributions

LJ, RJ, and H-LZ designed the study. LJ, HZ, and LH extracted the data. LJ, HZ, and RJ performed the statistical analysis and interpreted the data. LJ, HZ, L-HJ, and RJ drafted the manuscript. H-LZ critically revised the manuscript. All authors approved the final version for submission and publication.

## Footnotes

1
https://www.cdc.gov/nchs/nhanes/

2
https://wwwn.cdc.gov/Nchs/Nhanes/2011-2012/DEMO_G.htm#RIDAGEYR

3
https://wwwn.cdc.gov/Nchs/Nhanes/2013-2014/CFQ_H.htm

4
http://www.empowerstats.com

5
https://wwwn.cdc.gov/nchs/nhanes/tutorials/module3.aspx
